# Supplementary material for: JIP3 links lysosome transport to regulation of multiple components of the axonal cytoskeleton
Source: Commun Biol. 2022 Jan 10;5:5. doi: 10.1038/s42003-021-02945-x (PMC8748971; doi:10.1038/s42003-021-02945-x)
Supplement: Supplementary file 1 — Supplemental Material [file 42003_2021_2945_MOESM1_ESM.pdf]

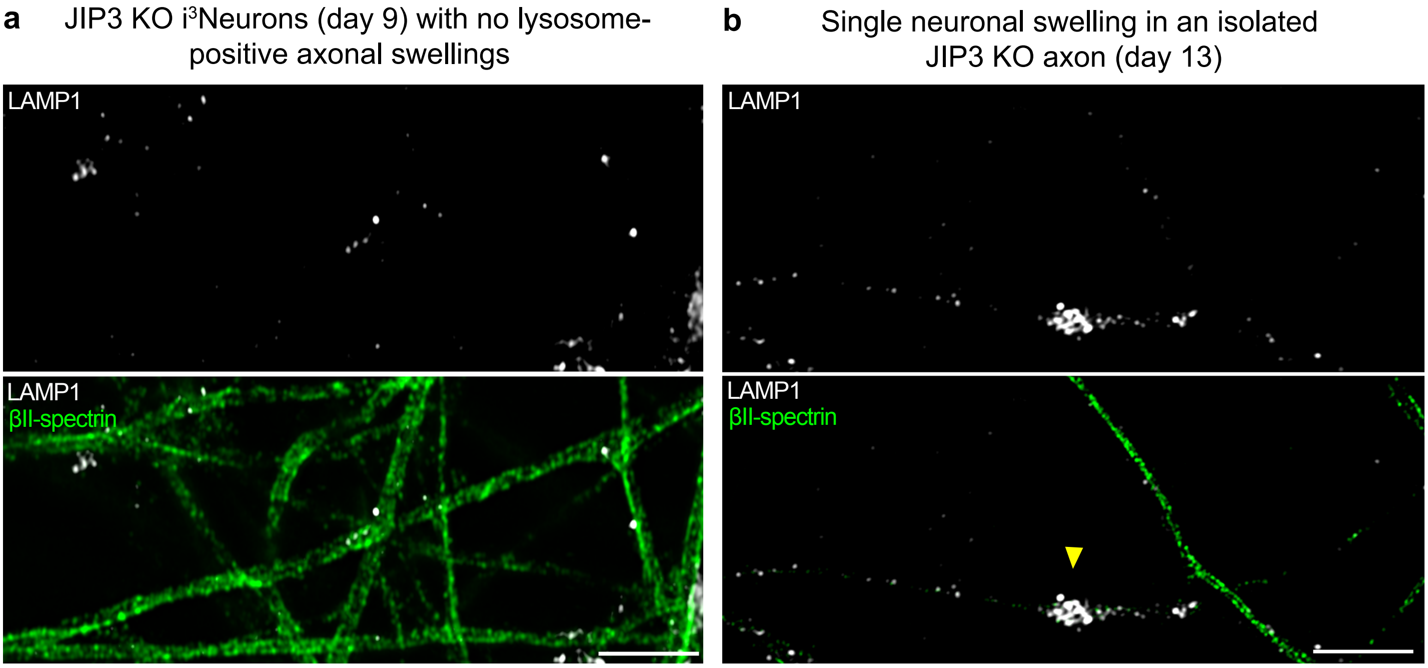


**Supplementary Figure 1: βII-spectrin organization in JIP3 KO i³Neurons**
(a) Airyscan microscopy images of young JIP3 KO i3Neurons (day 9) with no lysosome-positive swellings (LAMP1, white) display intact periodic membrane skeleton (βII-spectrin, green). (b) The global disruption of the periodic membrane skeleton shown in Figure 1H is also seen in JIP3 KO i³Neuron (day 13) with very sparse lysosome-positive axonal swellings. Lysosomes and the periodic membrane skeleton were labeled with LAMP1 and βII-spectrin antibodies, respectively. Scale bars, 5 μm.

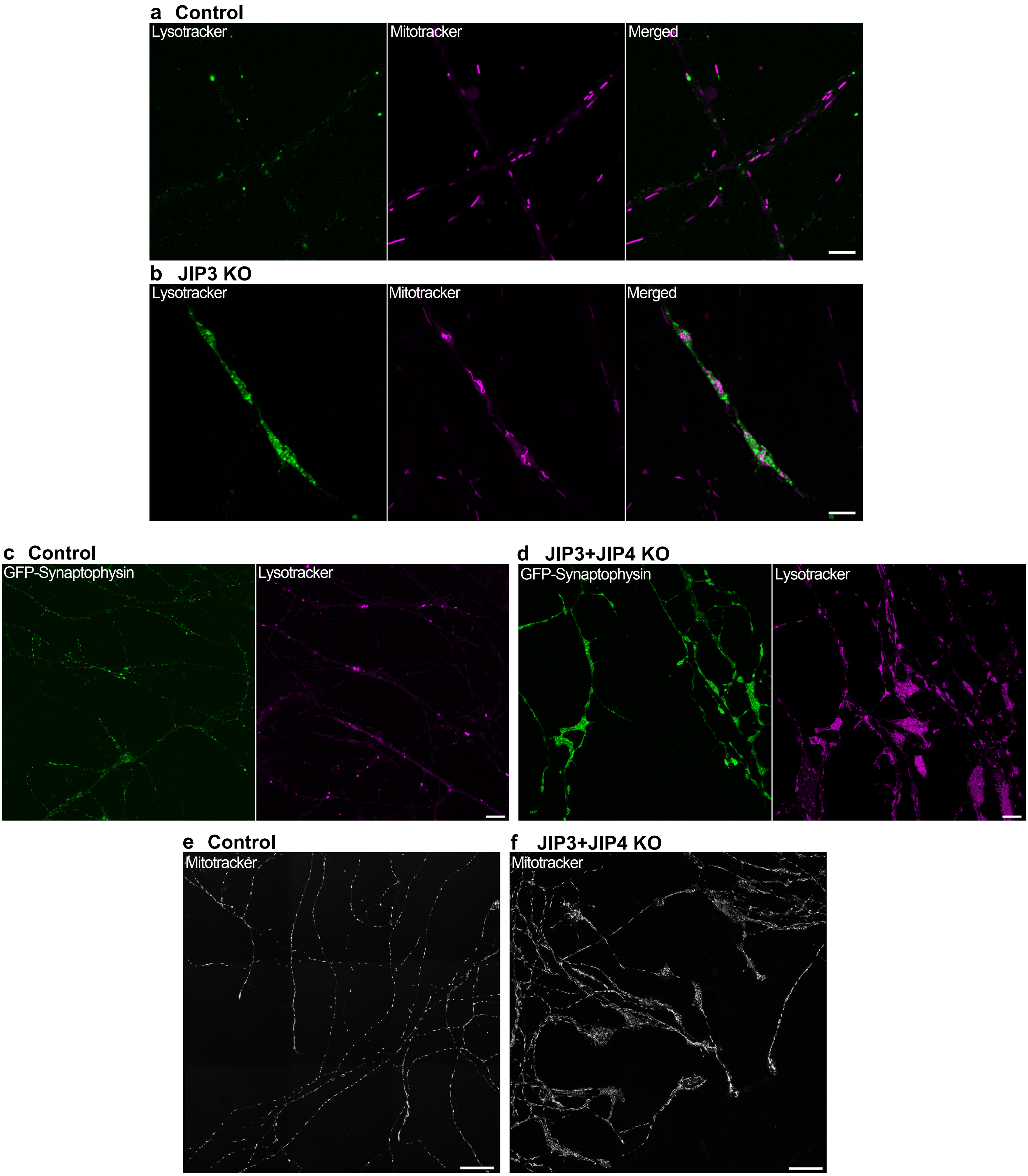


**Supplementary Figure 2: In addition to lysosomes, synaptic vesicles and mitochondria are also present within axonal swellings in the JIP3 KO and JIP3+JIP4 KO i$^3$Neurons**

(a and b) Representative Airyscan microscopy images of lysosomes (green) and mitochondria (magenta) in control (a) and JIP3 KO (b) i$^3$Neurons (day 15). Scale bars, 5 µm. Note that while lysosomes strongly accumulate in JIP3 KO i$^3$Neurons, mitochondria are also present in these lysosome-positive swellings, but to a much lesser degree. (c, d) Airyscan microscopy images of synaptic vesicles (green) and lysosomes (magenta, lysotracker) control and JIP3+JIP4 KO i$^3$Neurons respectively (10 days of differentiation). (e,f) Mitochondria localization in control versus JIP3+JIP4 KO i$^3$Neurons (Airyscan images, day 10). Scale bars, 10 µm.

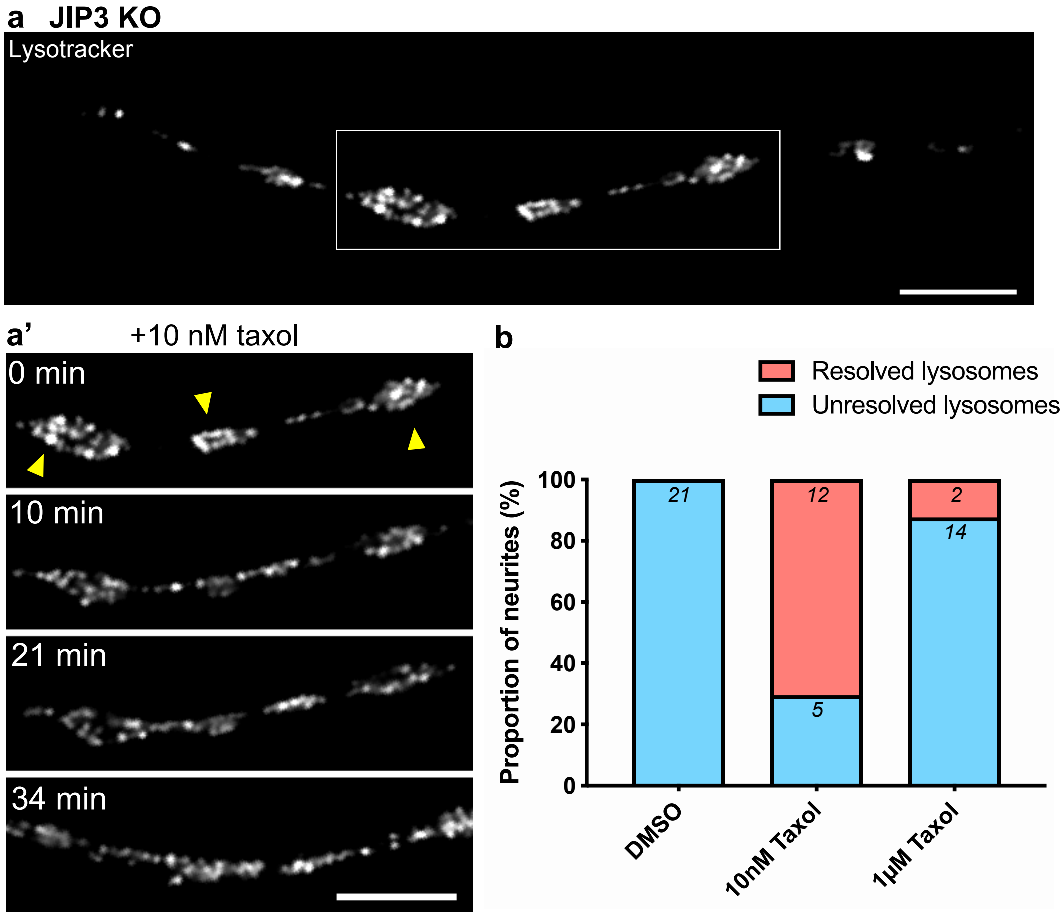


**Supplementary Figure 3: Effect of taxol on axonal swelling in JIP3 KO i³Neurons**
(a) Representative Airyscan microscopy image of JIP3 KO i³Neuron with lysosome-positive axonal swellings (lysotracker, white). Scale bar, 5 μm. (a') Images from the boxed area in (a) at the indicated time points show dispersion of lysosomes from focal swellings (yellow arrowheads) when treated with low doses of taxol (10nM). Scale bar, 2 μm. (b) Percentage of neurites showing dispersion of lysosomes from focal swellings in JIP3 KO i³Neurons in the indicated conditions (pooled from at least three independent experiments with ≥3 swellings analyzed per experiment).

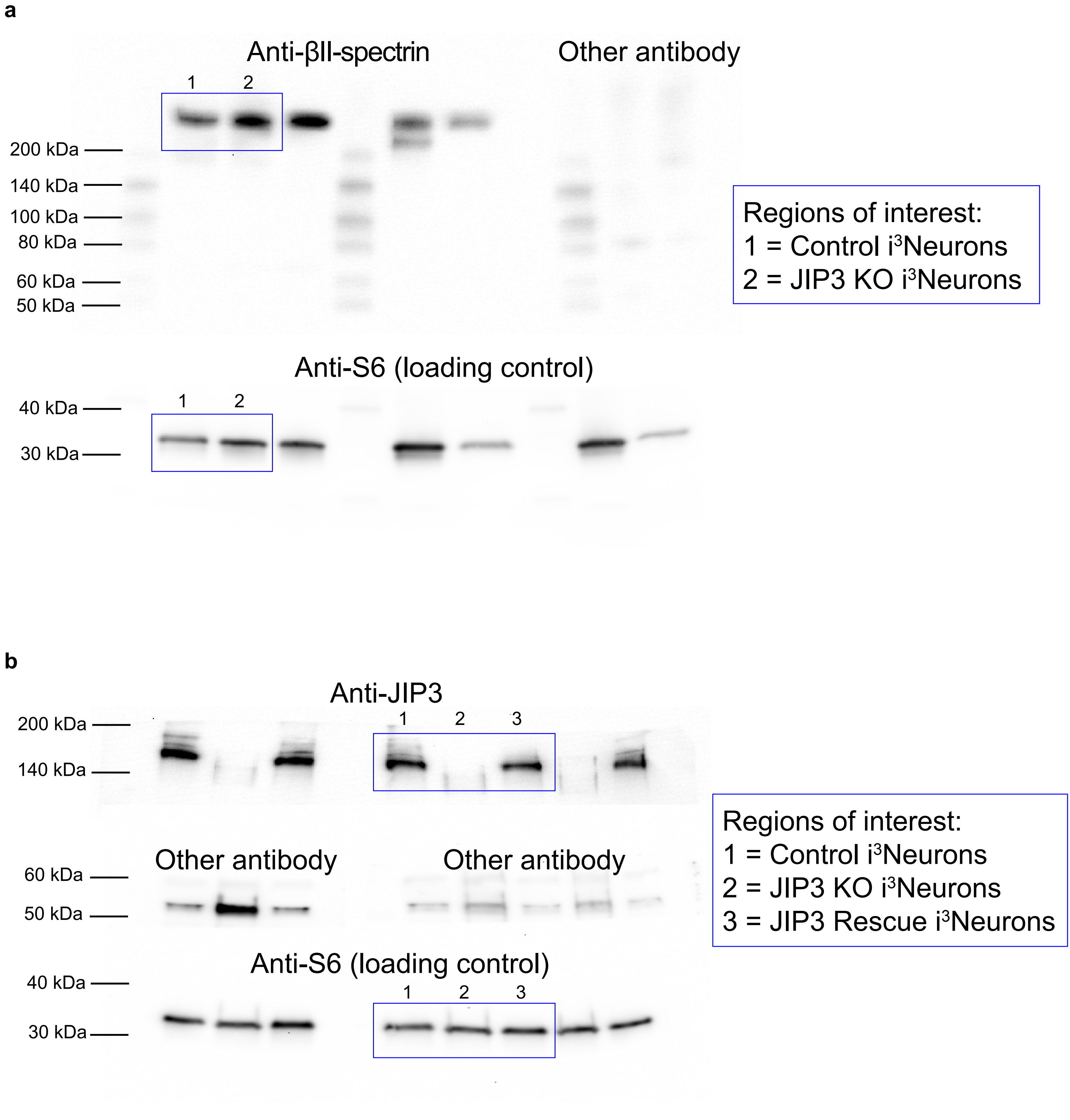


**Supplementary Figure 4:** Uncropped images of western blots of i$^3$Neurons showed in (a) Figure 1k and (b) Figure 5a. See detailed description in the legends of the corresponding figures. The boxed areas (blue frames) show the cropped images.
