# Supplementary material for: JIP3 links lysosome transport to regulation of multiple components of the axonal cytoskeleton
Source: Commun Biol. 2022 Jan 10;5:5. doi: 10.1038/s42003-021-02945-x (PMC8748971; doi:10.1038/s42003-021-02945-x)
Supplement: Supplementary file 2 — Description of Additional Supplementary Files [file 42003_2021_2945_MOESM2_ESM.pdf]

Description of Additional Supplementary Files

File name: Supplementary Movie 1.
Description: Dynamic formation and resolution of axonal swellings in JIP3 KO i3Neurons.
JIP3 KO i 3Neurons (day 15) stably expressing LAMP1-GFP were labeled with SiR tubulin to mark lysosomes and microtubules, respectively. Images were acquired at 5-minute intervals over a period of 12 hours using Airyscan microscopy. Scale bar, 5 µm. Display rate is 5 frames/sec.

File name: Supplementary Data 1-14.
Description:

Supplementary Data 1: Source file for Figure 1c.

Supplementary Data 2: Source file for Figure 1f.

Supplementary Data 3: Source file for Figure 1i.

Supplementary Data 4: Source file for Figure 1j.

Supplementary Data 5: Source file for Figure 1l.

Supplementary Data 6: Source file for Figure 2c.

Supplementary Data 7: Source file for Figure 2f.

Supplementary Data 8: Source file for Figure 2i.

Supplementary Data 9: Source file for Figure 3b.

Supplementary Data 10: Source file for Figure 3c.

Supplementary Data 11: Source file for Figure 3f.

Supplementary Data 12: Source file for Figure 4d.

Supplementary Data 13: Source file for Figure 5e.

Supplementary Data 14: Source file for Figure 5i.
