# Supplementary material for: JIP3 links lysosome transport to regulation of multiple components of the axonal cytoskeleton
Source: Commun Biol. 2022 Jan 10;5:5. doi: 10.1038/s42003-021-02945-x (PMC8748971; doi:10.1038/s42003-021-02945-x)
Supplement: Supplementary file 5 — Reporting Summary [file 42003_2021_2945_MOESM5_ESM.pdf]

nature portfolio

Corresponding author(s): COMMSBIO-21-0333C

Last updated by author(s): Nov 8, 2021

Reporting Summary

Nature Portfolio wishes to improve the reproducibility of the work that we publish. This form provides structure for consistency and transparency in reporting. For further information on Nature Portfolio policies, see our Editorial Policies and the Editorial Policy Checklist.

Statistics

For all statistical analyses, confirm that the following items are present in the figure legend, table legend, main text, or Methods section.

n/a	Confirmed	
☐	☒	The exact sample size (*n*) for each experimental group/condition, given as a discrete number and unit of measurement
☐	☒	A statement on whether measurements were taken from distinct samples or whether the same sample was measured repeatedly
☐	☒	The statistical test(s) used AND whether they are one- or two-sided<br>*Only common tests should be described solely by name; describe more complex techniques in the Methods section.*
☐	☒	A description of all covariates tested
☒	☐	A description of any assumptions or corrections, such as tests of normality and adjustment for multiple comparisons
☐	☒	A full description of the statistical parameters including central tendency (e.g. means) or other basic estimates (e.g. regression coefficient) AND variation (e.g. standard deviation) or associated estimates of uncertainty (e.g. confidence intervals)
☐	☒	For null hypothesis testing, the test statistic (e.g. *F*, *t*, *r*) with confidence intervals, effect sizes, degrees of freedom and *P* value noted<br>*Give P values as exact values whenever suitable.*
☒	☐	For Bayesian analysis, information on the choice of priors and Markov chain Monte Carlo settings
☒	☐	For hierarchical and complex designs, identification of the appropriate level for tests and full reporting of outcomes
☒	☐	Estimates of effect sizes (e.g. Cohen's *d*, Pearson's *r*), indicating how they were calculated

Our web collection on statistics for biologists contains articles on many of the points above.

Software and code

Policy information about availability of computer code

Data collection	The ZEN Black imaging software (Zeiss) was used to collect confocal and Airyscan images. The LAS X software (Leica Microsystems) was used to collect STED images, and the final images were deconvolved using Huygens deconvolution software (Huygens Essentials, Scientific Volume Imaging). All the softwares are commercially available. The softwares were coupled with the respective microscopes described in the Materials and Method section of the manuscript.
Data analysis	ImageJ (open source image processing software) Ver 2.1.0/1.53c was used for image viewing and processing. Percentage of neurites were quantified using the FIJI plugin “NeuronJ” and/or the FIJI segmented lines + ROI manager tool to determine the total number of neurites. For quantification of βII-spectrin intensity, neurites were semi-automatically traced and quantified using “NeuronJ” to determine the mean fluorescence value of the traced segments.<br><br>Western blot data were processed using Image Lab software (Bio-Rad) and quantified using the “Gels” ImageJ plugin. The methods for statistical analysis and sizes of the samples (n) are specified in the results section or figure legends for all of the quantitative data. Student's t test or Mann-Whitney test was used when comparing two data sets. Differences were accepted as significant for P < 0.05. Prism version 9 (GraphPad Software) was used to plot, analyze and represent the data.

For manuscripts utilizing custom algorithms or software that are central to the research but not yet described in published literature, software must be made available to editors and reviewers. We strongly encourage code deposition in a community repository (e.g. GitHub). See the Nature Portfolio guidelines for submitting code & software for further information.

Data

Policy information about availability of data

All manuscripts must include a data availability statement. This statement should provide the following information, where applicable:

- Accession codes, unique identifiers, or web links for publicly available datasets
- A description of any restrictions on data availability
- For clinical datasets or third party data, please ensure that the statement adheres to our policy

All data generated or analysed during this study are included in this published article (and its supplementary information files). Raw datasets generated during and/ or analysed during the current study are available from the corresponding author on reasonable request.

Field-specific reporting

Please select the one below that is the best fit for your research. If you are not sure, read the appropriate sections before making your selection.

☒ Life sciences ☐ Behavioural & social sciences ☐ Ecological, evolutionary & environmental sciences

For a reference copy of the document with all sections, see nature.com/documents/nr-reporting-summary-flat.pdf

Life sciences study design

All studies must disclose on these points even when the disclosure is negative.

Sample size	Sample size were determined based on whether data is reproducible in independent sets of experiments, typically three independent experiments are used to determine the reproducibility of data.
Data exclusions	No data were excluded from the analyses.
Replication	All attempts with replication were successful, based on the observed phenomena (e.g. microtubule loop formation, disruptions to the spectrin network). Data are quantified each time, hence the repeatability can be assessed.
Randomization	Not applicable
Blinding	Blinding is not possible. The investigators prepared both control and treated samples at the same time for each independent set of experiments

Reporting for specific materials, systems and methods

We require information from authors about some types of materials, experimental systems and methods used in many studies. Here, indicate whether each material, system or method listed is relevant to your study. If you are not sure if a list item applies to your research, read the appropriate section before selecting a response.

Materials & experimental systems

n/a	Involved in the study	
☐	☒	Antibodies
☐	☒	Eukaryotic cell lines
☒	☐	Palaeontology and archaeology
☒	☐	Animals and other organisms
☒	☐	Human research participants
☒	☐	Clinical data
☒	☐	Dual use research of concern

Methods

n/a	Involved in the study	
☒	☐	ChIP-seq
☒	☐	Flow cytometry
☒	☐	MRI-based neuroimaging

Antibodies

Antibodies used	Immunofluorescence experiments: anti-α-tubulin (Sigma, catalogue no. T6199, dilution 1:500); anti-LAMP1 (Cell Signaling Technology, catalogue no. 9091, dilution 1:500) or (Developmental Studies Hybridoma Bank, clone 1D4B, dilution 1:500); anti-βII-spectrin (BD Transduction Laboratories, Clone 42/B-Spectrin II, dilution 1:250); anti-non muscle heavy chain of myosin-IIA (Sigma, catalogue no. M8064, dilution 1:500); anti-acetyl α-tubulin (Cell Signaling Technology, catalogue no. 5335, dilution 1:250); anti-detyrosinated α-tubulin (Abcam, catalogue no. ab48389, dilution 1:250); anti-tyrosinated α-tubulin (Millipore Sigma, clone YL1/2, dilution 1:250).<br>Western blot experiments: anti-JIP3 (Novus Biologicals, catalogue no. NBP1-00895, dilution 1:500); anti-S6 Ribosomal Protein (S6, Cell Signaling Technology, catalogue no. 2217, dilution 1:2500); anti-βII-spectrin (BD Transduction Laboratories, Clone 42/B-Spectrin

	II, dilution 1:1000).
Validation	Antibody validation was provided by manufacture's website (blot or cell images) and/or data provided in the manuscript.

Eukaryotic cell lines

Policy information about cell lines

Cell line source(s)	Human iPSCs (WTC11 line) harboring doxycycline-inducible Ngn2 to facilitate neuronal differentiation were received from Michael Ward (NINDS, NIH, USA). Information about the cell line is described in Fernadopulle et al, 2018 and in the Materials and Method section of this manuscript.
Authentication	Cell line were authenticated based on morphology and efficiency of neuronal differentiation. Genome editing was confirmed by sequencing of the target locus. Only cultures of low passages were used.
Mycoplasma contamination	Cell lines tested negative for mycoplasma contamination.
Commonly misidentified lines (See ICLAC register)	No commonly misidentified cell lines were used.
